# Medicinal animals used in ethnoveterinary practices of the 'Cariri Paraibano', NE Brazil

**DOI:** 10.1186/1746-4269-7-30

**Published:** 2011-10-10

**Authors:** Wedson MS Souto, José S Mourão, Raynner RD Barboza, Lívia ET Mendonça, Reinaldo FP Lucena, Maine VA Confessor, Washington LS Vieira, Paulo FGP Montenegro, Luiz CS Lopez, Rômulo RN Alves

**Affiliations:** 1 Programa de Pós-Graduação em Ciências Biológicas (Zoologia), Departamento de Sistemática e Ecologia, Universidade Federal da Paraíba, 58059-970 João Pessoa, PB, Brazil; 2 Departamento de Biologia, Universidade Estadual da Paraíba, Avenida das Baraúnas, Bodocongó, 58109-753 Campina Grande, PB, Brazil; 3 Departamento de Fitotecnia e Ciências Ambientais, Universidade Federal da Paraíba, 58397-000 Areia, PB, Brazil; 4 Mestrado em Biologia, Universidade de Coimbra, Colégio de S. Jerónimo, Largo de D. Dinis, Apartado 3026, 3001-401, Coimbra, Portugal; 5 Departamento de Sistemática e Ecologia, Universidade Federal da Paraíba, 58059-970 João Pessoa, PB, Brazil

## Abstract

**Background:**

Zootherapy is important in various socio-cultural environments, and innumerous examples of the use of animal derived remedies can currently be found in many urban, semi-urban and more remote localities in all parts of the world, particularly in developing countries. However, although a number of ethnobiological inventories concerning the use of medicinal animals in human health care have been compiled in Brazil in recent years, zootherapeutic practices in ethnoveterinary medicine (EVM) are poorly described and neglected in favor of human ethnomedicine. In this sense, the purpose of this study was to describe the local zootherapeutic practices in ethnoveterinary medicine of semi-arid of NE Brazil (Caatinga biome) and to contribute to future research about the validation of the effects and side effects of these animal products

**Methods:**

The information obtained through semi-structured interviews was complemented by free interviews and informal conversations. A total of 67 people were interviewed (53 men and 14 women) about the use and commercialization of medicinal animals. To determine the relative importance of each local known species, their use-values (UV) were calculated. Diversity of species utilized was compared, between localities, using rarefaction curves and diversity estimate (Chao2)

**Results and Conclusions:**

A total of 44 animal species (37 vertebrates and 7 invertebrates), distributed among 6 taxonomic categories were found to be used to treat 30 different ailments in livestock and pets. The results of our surveys revealed a rich traditional knowledge of local residents about the use of animals in traditional veterinary medicine. Although it is gradually being discontinued, the perceived efficacy, economic and geographic accessibility were main reasons for popularity of zootherapy in studied areas.

## Background

Animal husbandry is an economic activity closely linked to the needs of local consumption and trade, which significantly influences the political, social and economic contexts in Latin America [1]. The occupation of Brazil by Europeans (especially in the northeastern region) starting in the first half of the 16^th ^century was marked by the transmigration of numerous elements of the European fauna and flora [2], including cattle, goats and horses [3]. The importance of livestock to the occupation and settlement of the Brazilian semi-arid region by European colonists was synthesized by Darcy Ribeiro [4] as follows: 'The first settlements raised cattle, goats and people: the cattle to sell, the goats to eat and the people to migrate (further inland)'.

Through the centuries, the local descendants of Amerindians, Africans, and Europeans learned to use the native natural resources of the Caatinga (dryland) biome but also imported resources from the Old World to use in treating illnesses or infirmities in themselves and their livestock. The adaptation of the various human groups to the rich biological resources generated invaluable local knowledge systems that include extensive information on animal uses in general and medicinally useful species in particular [5]. Ever since, animals or animal parts have been broadly used in Brazilian traditional medicine and have played a significant role in healing practices [6-8].

The use of medicinal animals is a recognized and traditional manner of treating as curatives or palliatives for many health problems (sometimes in association with plant species), depending on the cultural background and local knowledge of the users [9-14]. The World Health Organization (WHO) estimates that up to 80% of the world's more than six billion people rely primarily on animal and plant-based medicines [15]. Recent publications have shown the importance of zootherapy in various socio-cultural environments, and innumerous examples of the use of animal derived remedies can currently be found in many urban, semi-urban and more remote localities in all parts of the world, particularly in developing countries [13,14,16-21]. However, in spite of the worldwide prevalence of traditional medical practices, research on medicinal animals has often been neglected in comparison to medicinal plants [14,15]. Pieroni et al. [22], for example, points out that studies on drugs of animal origin are still rare in the scientific literature. While Calixto [23] recorded 3,722 published full paper on medicinal plants in Brazil, inventories of animal species used as medicine are still relatively rare in the country.

Although a number of ethnobiological inventories concerning the use of medicinal animals in human health care have been compiled in Brazil in recent years [5-7,13,20,24-26], zootherapeutic practices in ethnoveterinary medicine (EVM) are poorly described and neglected in favor of human ethnomedicine. In this country, but less than 20 animal species were recorded in local EVM [27,28]. These limited descriptions of the ethnoveterinary resources of Brazil are in stark contrast to the economic importance of livestock rearing in the country, and the lack of regular access to essential medicines can greatly hamper productivity.

Traditional veterinary medicine is very important in developing countries where conventional remedies for animal health care are inaccessible or unaffordable to poor rural farmers [29]. According to the United Nations Food and Agricultural Organization (FAO), the lack of drugs to treat diseases and infections results in losses of 30-35% in the breeding sector of many developing countries, where poor animal health remains the major constraint to increased production [30]. High costs and inaccessibility (together with other problems associated with western-style healthcare systems) have helped maintain traditional treatment practices in these countries and fostered research on this subject [31].

Much effort is needed in research and integration of the ethnoveterinary practices activities in developing countries [32]. In many native and local stock raising communities if not all, a considerable proportion of useful ethno-knowledge and traditional animal health care practices remain unknown to date, albeit their increased demand to be integrated into primary animal health care delivery systems for wider use by rural and periurban communities [33]. While ethnoveterinary practices can lead to their validation and eventually to better animal healthcare provision and enhanced living standards of the rural poor [34,35], there is a notable scarcity of studies of zootherapy in EVM [28]. In fact, a recent compilation of ethnozoological studies published in Brazil recorded 87 works about zootherapeutic practices; however, only four were predominantly focused on the use of animals in traditional veterinary medicine [36].

The use of animals for medicinal purposes is part of a body of traditional knowledge which is increasingly becoming more relevant to discussions on conservation biology, public health policies, sustainable management of natural resources, biological prospection and patents [15,37]. In this perspective, the present survey was undertaken to document information about local animals used in veterinary medications by livestock raisers and rural populations in two municipalities located in Paraiba State, NE Brazil. The purpose of this study was (1) to describe the local zootherapeutic practices in EVM, (2) to insert the faunal resources explored for medicinal purposes in local EVM in the Brazilian Zootherapy databank, currently being developed in the Center of Ethnobiology and Ethnoecology, State University of Paraiba, Brazil, and (3) to contribute to future research about the effects and possible side effects of these animal products.

## Methods

### Study sites

**Figure 1:**
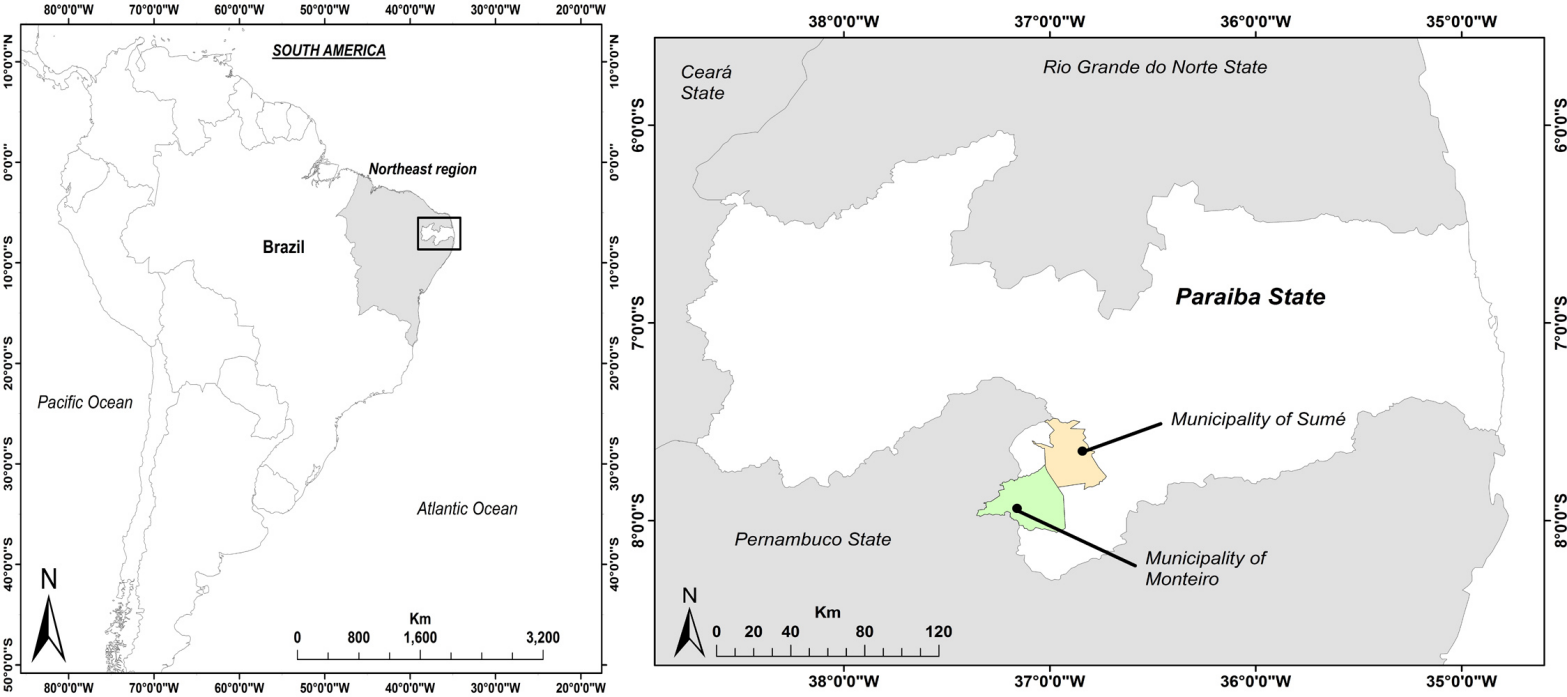
**Map showing the studied area**.

The present study analyzed data gathered during fieldwork in two municipalities in the West Cariri micro-region, Paraiba State, in the semi-arid region of Northeastern Brazil (Figure 1). Generally, human communities in the surveyed areas represent a mixture of native Amerindians, Europeans and Africans [38].

#### Municipality of Monteiro

The municipality of Monteiro (07° 53' 22" S and 37° 07' 12" W) covers an area of approximately 986 km^2 ^[38-40] within the 'drought polygon' of NE Brazil (an area that extends from northern Minas Gerais State and covers almost the entire northeastern part of the country). The regional climate is semi-arid with an annual rainfall of 432 mm (limited to a rainy season between January and April), with an average annual temperature of 28°C [41]. The vegetation of this semi-arid region (Caatinga biome) is typically composed of deciduous shrub/arboreal species and many of the plants have strong thorns.

The total population of the municipality is approximately 30,000, with 16,000 (53%) inhabitants in urban areas, and 14,000 (47%) residents in the rural zone [42]. This population has a medium Human Development Index level (0.603) [42]. The principal economic activities of the municipality are subsistence agriculture (mainly sweet potatoes, beans and cassava) and livestock breeding, including cattle (19,600 head), goats (30,000), and sheep (11,000). The municipality of Monteiro is the principal producer of goats and sheep in Paraiba State [38] and it is the most important economical center of the Cariri of Paraiba Amerindians. Animals (including wild animals) are commonly kept as pets in this locality.

Local interviewees ranged in age from 25 to 88 years (average 54). In terms of schooling, 43.75% (n = 14) of the interviewees were illiterate or semi-illiterate, while only 9.37% (n = 3) had attended school for 8 years (completing what is known in Brazil as 'ensino fundamental' - Elementary school). Most interviewees (53.12%, n = 17) had a monthly income between US$ 241.00 and $482.00).

#### Municipality of Sumé

The municipality of Sumé (6° 45' 28'' S × 36° 28' 15'' W) occupies an area of approximately 840 Km^2 ^[38,40] (Figure 1). The climate there is semi-arid with an annual rainfall of 695 mm that is limited to a rainy season between January and April [43]. The local vegetation is composed of spiny deciduous and semi-deciduous species characteristic of this semi-arid Caatinga region. Sumé had a population of 16,456 inhabitants in 2007, with 10,887 (66.1%) living in the urban zones, and 5,569 in rural areas (33.9%) [38]. The local inhabitants have a medium Human Development Index (0.658) [42]. Similar to Monteiro, the main economic activities in this municipality consist of subsistence agriculture (mainly sweet potatoes, beans and cassava) and livestock husbandry (8,600 head of cattle; 17,500 goats, and 6,100 sheep) [38].

The ages of the interviewees in Sumé ranged from 18 to 83 years (mean: 52.7); 45.71% (n = 16) of the interviewees were illiterate or semi-illiterate, 40% (n = 14) had attended school for less than eight years, while only five people (14.87%) had studied for more than eight years. As in Monteiro, most interviewees (59.37%, n = 19) had an income between US$ 241.00 and 482.00.

### Procedures

Field research was conducted from October 2008 to April 2009. The information obtained through semi-structured interviews was complemented by free interviews [44] and informal conversations. Prior informed consent was obtained for all interviews conducted. In the semi-structured interviews, the interviewees were requested to indicate for each animal: its local name; parts used as medicine; the ailments treated with the remedy; preparation and usage; use-restrictions; adverse effects; spiritual aspects linked to its use; whether live or dead animals were used; how the animals were obtained; storage conditions; collection sites; materials used to collect the animals; efficacy of the remedies; how this knowledge was acquired by the interviewees themselves; their reliance on animal-based remedies; and why they used animal-based remedies in ethnoveterinary practices. Interviews were held in both urban and rural settings of the municipalities studied. Before each interview, the interviewers asked their informants for permission to record the conversations and to take photographs. The ideal length of each interview was at least 40 minutes.

We interviewed 67 local residents (53 men and 14 women) about the use and commercialization of medicinal animals, and were distributed as follows: Municipality of Monteiro (n = 32; 24 men and 8 women), Municipality of Sumé (n = 35; 29 men and 6 women). All of the interviewees raised (or had risen) livestock. Verification tests were performed to determine the consistency and validity of the responses (for analysis and data control) by repeating details of the interviews in synchronic situations [45].

The zoological material was identified with the aid of specialists by: (a) examination of voucher specimens donated by the interviewees; (b) photographs of the animals or their parts, taken during interviews; (c) identification of vernacular names by taxonomists familiar with the fauna of the study areas. The voucher specimens and/or photographs were deposited at the Department of Systematics and Ecology, Federal University of Paraiba, Paraiba State, Brazil.

For the data analysis, the use-value (adapted from Phillips et al. [46] by Rossato et al. [47]), as a quantitative method demonstrating the relative importance of each species, was calculated as:

UV=ΣU∕n

Where UV is the use-value of a species, U the number of citations per species; and n is the number of informants. The use-value of each species is based solely on the importance attributed by the informants themselves and does not depend on any evaluation of the researcher [14,48].

We calculated the collector curves for both municipalities where the × axis was the number of individuals interviewed and the Y one was the number of animal species utilized for veterinary purposes. Collector curves were randomized 1000 times and the average values were calculated using the software EstimateS^© ^version 8.2 [49]. The same software was used to calculated an estimate of diversity (Chao2) projecting the total number of species utilized at each area. Chao2 was chosen because it is applied to incidence data. To input data in EstimateS^© ^we create a matrix interviewees (lines) × type of species (columns) for each municipality. In developing of matrix, we assigned the value 1 for each species mentioned by an interviewee and 0 for those which he did not mention. Both collector curves and diversity estimates were used to compare the diversity of animal species utilized between municipalities [50].

## Results and Discussion

### Zootherapeutic species used in ethnoveterinary medicine of 'Cariri Paraibano': an overview

According to 92% of local interviewees, ethnoveterinary knowledge was transmitted orally from generation to generation, especially from father to child and constitutes part of the culture of the people who live in the Caatinga region. In some cases, however, the obtained ethnoveterinary knowledge is derived from friends or neighbors.

The data obtained during the field surveys is summarized in Table 1. Forty four species (37 vertebrates and 7 invertebrates) were found to be used for medicinal or magic/religious purposes in ethnoveterinary medicine in the municipalities of Monteiro and Sumé. These species were distributed among at least 32 zoological families. The taxonomic group with the largest number of species were the mammals (with 19 species), followed by reptiles (8), birds and insects (both with 7 species). Other groups mentioned by the interviewees were fishes (2) and amphibians (1). The predominance of vertebrates reported in our study is similar to other studies of the use of animal-based remedies in human ethnomedicine [6,7,24,51-56]. This total is significant since it represents 16% of the entire registered traditional Brazilian zootherapeutic *pharmacopoeia*, which is composed of at least 290 animal species [26].

**Table 1:** Zootherapeutics resources used in Ethnoveterinary medicine of Cariri microregion, Paraiba State, Brazil

Family/Species/ Local name	Local of citation	Number of times mentioned	Use-value (VU)	Part used and way of administration	Disease (or illness)	Animal(s) treated
**INSECTA**						
**Apidae**						
*Apis mellifera *(Linnaeus, 1758) - Italian honey bee, "abelha italiana"	MO, SU	3	0.04	Honey (1)	**Eye problems, especially blindness and inflammations**	ca, eq, go, sh
				Honey (2)	Colds in cattle	ca
*Melipona subnitida *(Ducke, 1910) - jandaíra bee, "Abelha Jandaíra"	MO, SU	3	0.04	Honey (1)	Eye problems, especially blindness and inflammations	Domestic animals in general
				Honey (2)	**Colds in cattle**	ca
*Partamona seridoensis *Pedro & Camargo, 2003 - "abelha cupira", cupira bee	MO, SU	35	0.52	Honey (1)	Eye problems, especially blindness and inflammations; **swellings**, **dermal inflammations**, **'estrepes' **(suck a splinter out of skin), **wounds**, **furunculosis**, **lesions**	Domestic animals in general, mainly ca, go, sh, eq.
				Honey (2)	Colds in cattle	ca
				Honey (2)	Chickens' gogo (infectious coryza, a type of cold)	ch
				'Saburá' (3)	'Mother's body' (uterine prolapse)	ma, co, go, sh
*Scaptotrigona *sp. - "abelha canudo"	SU	1	0.01	Honey (1)	Eye problems, especially blindness and inflammations	Domestic animals in general
**Bothriuridae**						
*Bothriurus asper *Pocock, 1893 - black scorpion	MO	6	0.09	Sting (4)	Dermal nodules and furunculosis	ca, go, sh
**Buthidae**						
*Rhopalurus rochai *(Borelli, 1910) - "Escorpião amarelo do sertão"	MO	7	0.10	Sting (4)	Dermal nodules and furunculosis	ca, go, sh
**Termitidae**						
*Nasutitermes corniger *(Motschulsky, 1855) - termite black	MO	6	0.09	Whole animal (2)	Chickens' gogo (infectious coryza, a type of cold)	ch, hg
**FISHES**						
**Electrophoridae**						
*Electrophorus electricus *(Linnaeus, 1766) - electric eel	MO, SU	2	0.03	Fat (1)	**Wounds**	ca, ct, do, go, ho, sh, eq
**Erythrinidae**						
*Hoplias malabaricus *(Bloch, 1794) - Trahira, "traíra"	MO	4	0.06	Fat (1)	Lesions in eyes and hooves of cattle	ca
**AMPHIBIANS**						
**Bufonidae**						
*Rhinella schneideri *(Werner, 1894) - Cururu toad, "sapo cururu" ^LC^	SU	11	0.16	Viscera (1)	'Esponja de cavalo' (Dermal wounds brought about by infestation of larvae of *Habronema muscae*)	eq
				Fat (1)	**Wounds**, **'estrepes' **(suck a splinter out of skin), lesions	Domestic animals in general
**REPTILES**						
**Alligatoridae**						
*Caiman latirostris *(Daudin, 1801) - Cayman, "jacaré-do-papo-amarelo" ^LC^	MO	1	0.01	Leather (5), fat (1)	Wounds, 'estrepes' (suck a splinter out of skin)	ca, ct, do, go, ho, sh, eq
"Black alligator" -Unidentified species	MO	1	0.01	Leather (5), fat (1)	Wounds, 'estrepes' (suck a splinter out of skin)	ca, ct, do, go, ho, sh, eq
**Chelidae**						
*Phrynops geoffroanus *(Schweigger, 1812) - Geoffroy's side-necked turtle, "cágado"	MO, SU	30	0.45	Fat (1)	Wounds, 'estrepes' (suck a splinter out of skin), **ear problems**, inflammations, dermal nodules, furunculosis, burns	Some animal, mainly ca, ct, do, go, ho, sh, eq
				Fat (3)	'Mother's body' (uterine prolapse)	Some livestock, mainly cattle
**Iguanidae**						
*Iguana iguana *(Linnaeus, 1758) - Common Green Iguana, "Camaleão	MO, SU	9	0.13	Fat (2)	Throat problems	ca
				Fat (1)	**Wounds, 'estrepes', eye problems**	ca, ct, do, go, ho, sh, eq
**Teiidae**						
*Tupinambis merianae *(Duméril & Bibron, 1839) - Lizard teju, "tegu", "tejuaçú	MO, SU	48	0.72	Fat (1)	Burns, inflammations, **wounds**, **'estrepes'**, lesions, ear problems, **throat problems**, **swellings**, dermal nodules, furunculosis, **snake bite**, cracks in hooves of cattle, Eye problems, especially blindness	ca, ct, do, go, ho, sh, eq
				Fat (2)	Sore throat	ct, do, cv
				Fat (2)	Intestinal infections, snake bite	ca, ct, do, go, ho, sh, eq
**Order Testudines**						
"Sea turtle" - Espécie não identificada	MO	1	0.01	Fat (1)	Wounds	Domestic animals in general
**Testudinidae**						
*Chelonoidis carbonaria *(Spix, 1824) - Red-footed tortoise, "jabuti"	MO	1	0.01	Fat (1)	'Estrepes' (suck a splinter out of skin)	Domestic animals in general
**Viperidae**						
*Crotalus durissus *Linnaeus, 1758 - South American rattlesnake, "Cascavel"	MO, SU	44	0.66	Fat (1)	**Wounds**, 'estrepes', lesions, dermal nodules, furunculosis, **snake bite**; Eye problems, especially blindness and inflammations	ca, ct, do, go, ho, sh, eq
				Fat (6)	**Rheumatism**	ca, eq
				Fat (2)	Fever, **throat problems**	cv
				'Maracá' (rattle) (7)	Protect the cattle against snake bites	ca
**BIRDS**						
**Cariamidae**						
*Cariama cristata *(Linnaeus, 1766) - "sariema" ^LC^	MO	1	0.01	Fat (1)	Swellings	Domestic animals in general
**Cathartidae**						
*Coragyps atratus *(Bechstein, 1793) - Black vulture, "urubu", "urubu-preto" ^LC^	MO	1	0.01	Feather (8)	Tick fever	ca
**Corvidae**						
*Cyanocorax cyanopogon *(Wied-Neuwied, 1821) - White-naped Jay, "Pássaro cancão" ^LC^	MO, SU	2	0.03	Feather (8)	Tick fever	ca
				Whole animal (9)	**To protect livestock against 'evil eyes'**	ca, eq, go, pi
**Meleagrididae**						
*Meleagris gallopavo *Linnaeus, 1758 - turkey, "peru"	MO, SU	8	0.12	Fat (1)	**'Estrepes', lesions, wounds**	ca, ct, do, go, ho, sh, eq
**Phasianidae**						
*Gallus gallus domesticus *Linnaeus, 1758) - Domestic chicken, "Galinha"	MO, SU	8	0.12	Fat (1)	Inflammations, dermal nodules, **'estrepes'**, furunculosis, lesions, mastitis	ca, ct, do, go, ho, sh, eq
				Fat (2)	**Throat problems**	cv
				Fat (10)	"Oca" (Bovine gangrenous coryza)	ca
				Fat (6)	Rheumatism	ca, eq
				Eggs (1)	**Weakness**	cv
**Rheidae**						
*Rhea americana *(Linnaeus, 1758) - Greater rhea, "ema" ^NT^	MO, SU	5	0.07	Fat (1)	**'Estrepes' **(suck a splinter out of skin), **wounds, lesions, furunculosis**	ca, ct, do, go, ho, sh, eq
**Tinamidae**						
*Nothura maculosa cearensis *Naumburg, 1932 - Spotted Nothura, "Codorniz" ^LC^	MO, SU	22	0.33	Feather (11, 12)	**Snake bites**	Domestic animals in general
**MAMMALS**						
**Agoutidae**						
*Agouti paca *(Linnaeus, 1766) - Spotted paca, "paca" ^LC^	MO	1	0.01	Bile (2)	Snake bites	Domestic animals in general
**Bovidae**						
*Bos taurus *Linnaeus, 1758 - Domestic cattle, "Vaca"	MO, SU	16	0.24	Milk (13)	To treat intestinal worms (anthelmintic)	ca, ct, do, go, ho, sh, eq
				Milk (1)	Mastitis	co, go, sh
				Homemade butter (1)	Dermal nodules, burns, 'estrepes', **inflammations**, mastitis	ca, ct, do, go, ho, sh, eq
				Homemade butter (2)	Throat problems	cv
				Horn or skull (14)	To protect animals against 'evil eyes'	ca, ct, do, go, ho, sh, eq
*Capra hircus *Linnaeus, 1758 - "bode"	MO, SU	6	0.24	Fat (1)	Wounds	ca, ct, do, go, ho, sh, eq
				Homemade butter (1)	Dermal inflammation	ca, ct, do, go, ho, sh, eq
				Leather (7)	To protect animals against snake bites	ca, ho, go, sh
*Ovis aries *Linnaeus, 1758 - Ram, sheep, "Carneiro"	MO, SU	60	0.90	Fat/Castrated ram suet (1)	Wounds, 'estrepes', lesions, bone fractures, 'junta dura' (rheumatism), dermal nodules, inflammations, swellings	ca, ct, do, go, ho, sh, eq
				Castrated ram suet (10)	'Oca' (Bovine gangrenous coryza)	ca
				Castrated ram suet (6)	Rheumatism	ca, ho, ma
				Fat/Castrated ram suet (15)	'Caruara de bezerro' (omphaloarteritis)	cv
				Horn or skull (14)	To protect animals against 'evil eyes'	ca, ct, do, go, ho, sh, eq
				Leather (7)	To protect animals against snake bites	ca, go, ho, sh
**Canidae**						
*Canis lupus familiaris *Linnaeus, 1758 - Domestic dog, "cachorro"	MO	1	0.01	Head (1)	Retained placenta	co
*Cerdocyon thous *(Linnaeus, 1766) - Crab-eating fox, "raposa" ^LC^	MO, SU	47	0.70	Fat (1)	**Wounds**, '**estrepes'**, inflammations, lesions	Domestic animals in general
				Fat (2)	Throat problems	cv
				Tail (16), Leather (16)	'To protect animals against attacks by bats'	ca, go, sh, eq and mainly chickens
				Fat (17)	Respiratory problems	ca
				Suet (6)	**'Junta dura' (rheumatism)**	ca, ho, ma
				Fat (3)	'Mother's body' (uterine prolapse)	co
**Caviidae**						
*Cavia aperea *Erxleben, 1777 - "Preá" ^LC^	MO	1	0.01	Fat (1)	Furunculosis	ca, ct, do, go, ho, sh, eq
*Kerodon rupestris *(Wied-Neuwied, 1820) - Rock cavy, "Mocó" ^LC^	SU	3	0.04	Fat (1)	Spine problems	ca
				Meat (2)	**Weakness **in dogs and cats	ct, do
**Order Cetacea**						
"Baleia" - unidentified species	SU	1	0.01	Fat (1)	Wounds, 'estrepes'	ca, ct, do, go, ho, sh, eq
**Cervidae**						
*Mazama gouazoupira *(G. Fischer, 1814) - Gray brocket, "veado catingueiro"	MO	3	0.04	Horn (14)	To protect animals against 'evil eyes'	ca, eq, go, sh
				Fat (6)	'Junta dura' (rheumatism)	ca, eq, go, sh
				Fat (1)	Lesions	ca, eq, go, sh
**Dasypodidae**						
*Dasypus novemcinctus *(Linnaeus, 1758) - Nine-banded armadillo, "tatu galinha" ^LC^	MO	1	0.01	Fat (1)	Wounds	Domestic animals in general
*Euphractus sexcinctus *(Linnaeus, 1758) - Six-banded armadillo "tatu peba" ^LC^	MO	1	0.01	Fat (1)	Wounds	Domestic animals in general
**Felidae**						
*Leopardus tigrinus *(Schreber, 1775) - Little Tiger Cat, "gato-pintado" ^VU^	MO, SU	11	0.16	Fat (2)	Intestinal disorders	ca, ct, do, go, ho, sh, eq
				Fat (1)	'Estrepes' (suck a splinter out of skin)	ca, ct, do, go, ho, sh, eq
				Tail (16), Leather (16)	'To protect animals against attacks by bats'	ca, ct, do, go, sh, eq and mainly chickens
				Leather (5)	Swellings	Domestic animals in general
*Puma yagouaroundi *(É. Geoffroy Saint-Hilaire, 1803) - jaguarundi, "gato-do-mato vermelho", "gato-do-mato azul" ^VU^	MO, SU	14	0.21	Fat (2)	Intestinal disorders	ca, ct, do, go, ho, sh, eq
				Fat (1)	'Estrepes' (suck a splinter out of skin)	ca, ct, do, go, ho, sh, eq
				Tail (16), Leather (16)	'To protect animals against attacks by bats'	ca, ct, do, go, ho, sh, eq
				Leather (5)	Swellings	Domestic animals in general
**Hominidae**						
*Homo sapiens *Linnaeus, 1758 - human	MO	2	0.03	Urine (2)	To tame angry animals	ca, eq, go, sh
**Mustelidae**						
*Conepatus semistriatus *(Boddaert, 1785) - Striped hog-nosed skunk, "cangambá", "gambambá", tacaca ^LC^	MO, SU	2	0.03	Meat (2)	**Weakness **in dogs and cats	ct, do
				Bones (18)	**'Junta dura' (rheumatism)**	ca, ho, go
**Myrmecophagidae**						
*Tamandua tetradactyla *(Linnaeus, 1758) - Collared Anteater	SU	8	0.12	Leather (16)	'To protect animals against attacks by bats'	ca, ct, do, go, sh, eq and mainly chickens
**Procyonidae**						
*Procyon cancrivorus *(G. [Baron] Cuvier, 1798) - Crab-eating raccoon, "guaxinim" ^LC^	SU	3	0.04	Tail (16), Leather (16)	'To protect animals against attacks by bats'	ca, ct, do, go, sh, eq and mainly chickens
**Suidae**						
*Sus scrofa domesticus *Linnaeus, 1758 - domestic pig	MO, SU	3	0.04	Fat (1)	**Wounds**, bone fractures, mastitis	ca, ct, do, go, ho, sh, eq

*Study sites*: MO - Monteiro, SU - Sumé. *Animals treated*: ca - cattle, ch - chickens, co - cow, ct - cats, cv -calves, do - dogs, eq - equines, go - goats, hg - the helmeted guineafowl, ho - horses, ma - mares, pi - pigs, sh - sheeps. *IUCN Red List status*: LC - Least Concern, NT - Near Threatned, VU - Vulnerable. *Ways of administering the animal-based remedies*: (1) topical application, (2) oral application, (3) specialized technique where the uterus, after externalize, it is moistened and 'washed' with fat or other substance considered locally as healing and then carefully set back into the animal, (4) the sting of these animals are used on dermal pits in order to have a fast inflammation, followed by a quickly heal, (5) used as plaster, (6) applied on the joints of the members, (7) the rattlesnake's maracá or a piece of leather 'blessed' by a healer is hung on the neck of the livestock, (8) used as smoker, (9) Magical use, the animal must be bred to protect against evil eye, (10) local residents usually saw the cattle horny and fill in with fats/suet or other substances considered therapeutic, (11) Toast and triturate it and the resulting powder is applied on the affected area, (12) tea of the toasted powder, (13) mixed with american wormseed (mastruz) *Chenopodium ambrosioides L*. and taken as drink, (14) It should be placed in the corral to protect livestock against evil eye, (15) applied on the navel of the animal, (16) Magical-religious use, the leather or animal's tail is hanging on the neck or placed in the chicken pen entrance in order to protect the animals from bats attack, (17) used as expectorant, applied on the animal snout, (18) toast and triturate it and the resulting powder is ingested with food. Note: Diseases or conditions in bold are treated the same way in humans and animals.

**Figure 2:**
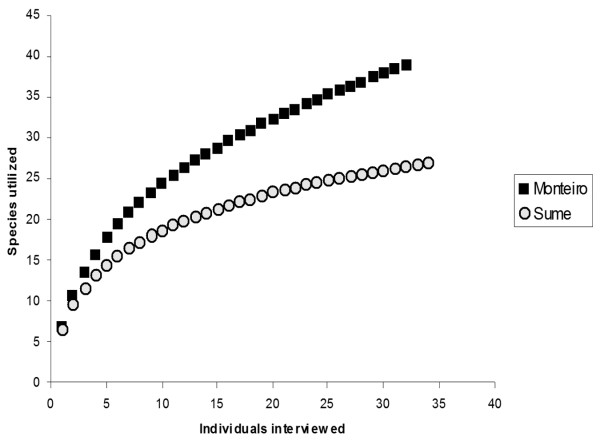
**Average rarefaction curves**. Average rarefaction curves, drawn from 1000 randomizations, for the number of animal species utilized for veterinary purpose in the municipalities of Monteiro and Sumé. This graph supports the hypothesis of a more diverse knowledge about animal uses for veterinary in Monteiro municipality.

Municipality of Monteiro presented a higher diversity of animals used for veterinary purposes, with 38 species cited compared to 27 from Sumé locality (Table 1). The higher diversity of animals used in Monteiro can be assessed visually from the collectors curves (Figure 2) where Monteiro's curve of species rise faster than Sumé one. The diversity estimator Chao2 also supports the hypothesis of Monteiro higher diversity, with Monteiro presenting an estimation of 62 species of animals utilized compared to the estimate of 41 species for Sumé municipality. A higher diversity of animal use found in Monteiro can be a result of historical and economical factors, since this municipality is a historical center of livestock husbandry and commerce in the region, a fact that could lead to a stronger and more diverse tradition in terms of folk veterinary.

In Brazil, most of the medicinal animal used in traditional medicine practices are collected from the wild [7,21]; this same trend was observed in the present study, where 36 (81%) of the species used in EVM of studies areas are wild caught. Nevertheless, some domestic animal species were also used to produce traditional medicines. These include the turkey (*Meleagris gallopavo *Linnaeus, 1758), domestic chicken (*Gallus gallus domesticus *Linnaeus, 1758), domestic cattle (*Bos taurus *Linnaeus, 1758), goats (*Capra hircus *Linnaeus, 1758), ram/sheep (*Ovis aries *Linnaeus, 1758), domestic dog (*Canis lupus familiaris *Linnaeus, 1758), domestic pig (*Sus scrofa domesticus *Linnaeus, 1758).

The links between human communities and the surrounding region became apparent when comparing the animal species used as remedies with the habitat types found near the study sites. Nearly all of the wild animals used were from terrestrial habitats (31 species) - a reflection of principal habitat types found in this semi-arid Caatinga biome. Similarly, Adeola [57] demonstrated that the utilization of wildlife in Nigeria was related to the ecological zone in which the people lived and to the relative abundance of the species in each zone. Our findings demonstrate the importance of local biodiversity in furnishing folk medicines, in agreement with Alves and Rosa [5] who observed that faunal composition, accessibility, and availability directly influence the types of zootherapeutic items used in any given region [14].

The use-value (UV) of the zootherapeutic resources was moderate. It ranged from 0.01 to 0.9. The most important medicinal species were the domestic ram *O. aries *(UV = 0.9), the teju lizard - *Tupinambis merianae *(Duméril & Bibron, 1839) (0.72), the crab-eating fox - *Cerdocyon thous *(Linnaeus, 1766) (0.7), the South American rattlesnake *- Crotalus durissus *Linnaeus, 1758 (0.66), the Cupira bee *- Partamona seridoensis *Pedro & Camargo, 2003 (0.52), Geoffroy's side-necked turtle *- Phrynops geoffroanus *(Schweigger, 1812) (0.45). Aside from Cupira bee, these species had been widely recorded in several studies conducted in Brazil on human ethnomedicine [5-7,13,20,21,24,25,58-61]. In Bahia State, for instance, the fat of *Ovis aries *is used to treat sprains [25] and in Pernambuco State the suet or fat of *O. aries *is used to treat joint problems, pains, rheumatism, and pits [62]. *Tupinambis merianae *has been cited as one of the principal animals used in human [13,14] and veterinary ethnomedicine [27,28] in NE Brazil. In this country, products derived from teju lizard have been indicated for treating 13 conditions in humans [7,13] and 8 in livestock [27,28,63].

The wild species with the highest use-values (*Tupinambis merianae, Cerdocyon thous, Crotalus durissus, Phrynops geoffroanus*) in local EVM that have been consistently reported in the studies of traditional medical practices in the northeastern Brazil constitute 'cultural keystone species' - a term that refers to those culturally important species that are associated with a group's cultural identity [64]. Garibaldi and Turner [65] listed several criteria used to identify cultural keystone species, including: (1) intensity, type, and multiplicity of uses; (2) names and terminologies incorporated into the local language, including their use as seasonal or phenological indicators; (3) roles in narratives, ceremonies, or symbolism; (4) persistence and memory of use in spite of cultural change, and (5) occupying a unique position in the culture.

**Figure 3:**
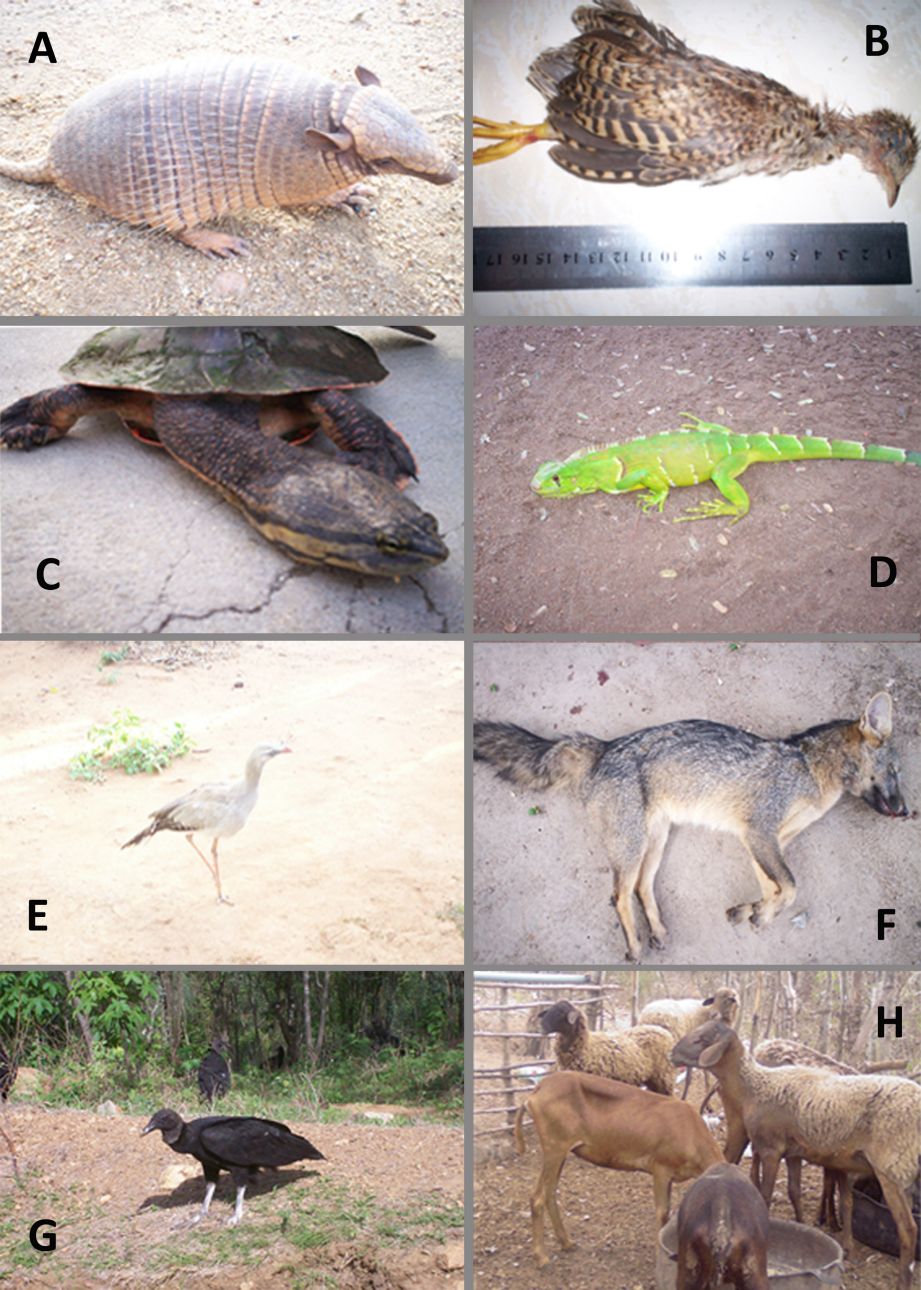
**Examples of animals used as medicine in ethnoveterinary practices of the 'Cariri Paraibano'**. (A) *Euphractus sexcinctus*, (B) *Nothura maculosa cearensis*, (C) *Phrynops geoffroanus*, (D) *Iguana iguana*, (E) *Cariama cristata*, (F) *Cerdocyon thous*, (G) *Coragyps atratus*, (H) *Ovis aries*. (A-G, photos by Wedson Souto; H, photo: Raynner Barboza).

The present study identified 20 species that had not been previously reported as being used in ethnoveterinary medicine in the semi-arid region of Brazil: the jandaíra bee - *Melipona subnitida *(Ducke, 1910), canudo bee - *Scaptotrigona *sp., termite black - *Nasutitermes corniger *(Motschulsky, 1855), electric eel - *Electrophorus electricus *(Linnaeus, 1766), The common wolfish - *Hoplias malabaricus *(Bloch, 1794), cururu toad - *Rhinella schneideri *(Werner, 1894), cayman - *Caiman latirostris *(Daudin, 1801), red-footed tortoise - *Chelonoidis carbonaria *(Spix, 1824), black vulture - *Coragyps atratus *(Bechstein, 1793), white-naped Jay - *Cyanocorax cyanopogon *(Wied-Neuwied, 1821), greater rhea - *Rhea americana *(Linnaeus, 1758), spotted-paca - *Agouti paca *(Linnaeus, 1766), 'preá' - *Cavia aperea *Erxleben, 1777, rock cavy - *Kerodon rupestris *(Wied-Neuwied, 1820), gray brocket - *Mazama gouazoupira *(G. Fischer, 1814), nine-banded armadillo - *Dasypus novemcinctus *(Linnaeus, 1758), small spotted cat - *Leopardus tigrinus *(Schreber, 1775), jaguarundi - *Puma yagouaroundi *(É. Geoffroy Saint-Hilaire, 1803), striped hog-nosed skunk - *Conepatus semistriatus *(Boddaert, 1785), Crab-eating raccoon - *Procyon cancrivorus *(G. [Baron] Cuvier, 1798). Additionally, the yellow scorpion-of-the-sertão - *Rhopalurus rochai *(Borelli, 1910), black scorpion - *Bothriurus asper *Pocock, 1893, Cupira bee - *Partamona seridoensis*, and the crested Cariama - *Cariama cristata *(Linnaeus, 1766) had not been previously reported in either Brazilian traditional human or veterinary ethnomedicine. Examples of medicinal animals are shown in Figure 3.

Six of the animals mentioned (black alligator, caiman, electric eel, sea turtle, spotted-paca, and whale) do not belong to the local fauna of the semi-arid region of northeastern of Brazil, and the products derived from them are therefore allochthonous zootherapeutics [66,67]. Two possible explanations for the use of exotic medicinal species are: (a) the existence of established trade routes for medicinal animals throughout the northern and northeastern Brazil [6,12,24,62,68,69] or (b) falsifications of traditional remedies (e.g., commercializing the fat of one species as being another) [70]. In relation to this, the use of whale fat is quite possibly an example of falsification, since Brazil has banned the hunting of marine mammals and, according to Brazilian law 7.643/87 and 9.605/98, hunting whales in Brazilian jurisdictional waters, as well as any kind of deliberate molestation such the pursuit of animals with vessels is prohibited [71].

Some of the medicinal animals used by the local populations in the present study are mentioned in medicinal literature from early colonial times, including: *Caiman latirostris, Crotalus durissus, Gallus gallus domesticus, Iguana iguana, Coragyps atratus*, and *Bos taurus *[8]. This observation corroborates with what Almeida [72] described as the 'high capability of reproduction of zootherapeutic practices in Brazil'. The persistent use of animal-based medicines suggests that they may contain substances of therapeutic value not yet known to formal science [14].

It is widely accepted that folk or traditional medicinal uses of biological resources (ethnomedical information) may indicate the presence of a biologically active constituent(s) in a species [73]. The prolonged and continuing use of a particular species in indigenous culture to treat certain ailments provides a presumed demonstration of efficacy [73]. In other words, folk or traditional medicinal uses represent 'leads' that could shortcut the discovery of modern medicines [5]. Notwithstanding, little attention has been given to the cultural, medical, economic, or ecological significance of zootherapeutic practices, however, even though Brazil's National Pharmaceutical Policy (Política Nacional de Medicamentos, Portaria no. 3916/98) specifies that 'the support of research designed to examine the therapeutic potential of the national flora and fauna, with emphasis on the certification of their medical properties, should be continued and expanded' [7].

Unfortunately, many aspects of traditional medical practices have not been described and there are tantalizing gaps in our knowledge of this subject. In many cases, traditional native medical practices have been either discontinued or greatly modified, and some traditional knowledge has been lost forever [74]. Modern health care in rural areas of Paraiba State is characterized by deficiencies in infrastructure, qualified personnel, medicines, and veterinary medicines [27,75,76], and the use of medicinal animals and plants (which are accessible and relatively inexpensive) is currently an important component of local health care for both humans and animals. Nevertheless, the gradual expansion of western allopathic medicine in recent years has discouraged zootherapeutic practices in traditional veterinary medicine in the semi-arid region of Paraiba State. This situation is confirmed by the fact that 95.5% (n = 64) of the interviewees indicated that their descendants (and local youths) were no longer interested in learning ethnoveterinary practices.

It is important to note that in addition to the perceived efficacy of animal-based remedies their popularity is influenced by cultural factors, and the relationships between humans and biodiversity (in the form of zootherapeutic species) are conditioned by the social and economic relations between humans themselves [6]. Although there are indications that local ethnoveterinary knowledge is being gradually being lost, some interviewees (n = 28) reported that the difficulties in finding commercial veterinary products and the high prices of these medications were the principal reasons that animal-derived medicines were still included in local veterinary practices. The financial limitations of many farmers make ethnoveterinary medicine a viable option for treating many infirmities afflicting their animals. Remedies that livestock keepers can prepare themselves from natural materials will cost less than buying therapeutic preparations ready-to-use, but the latter may be much cheaper than equivalent allopathic alternatives [77]. In terms of medicinal plants, several authors have found worldwide use-preferences influenced by the low prices of traditional medicines in local veterinary systems [34,78-80]. In the High Andes of Peru, for example, gathering plants and preparing homemade treatments for an average-sized family flock required only four hours of labor [81]. In contrast, the equivalent commercial product would cost a family about US$9 - in an area where daily wages are well under US$1 [81]. A locally processed herbal wound-powder in Sri Lanka was found to be as effective as a commercial product, but cost 80-90% less [82]. Our data is also consistent with Alves and Rosa [5,59] who found preferences for the use of animal-based medicines in low-income families in NE Brazil.

Given the importance of recording ethnoveterinary practices both for active-principal validation and for analysis of risks and impacts, we examined here how and why zootherapeutics species were used in EVM in the study areas and how they were obtained.

### Ways of preparing and administering of animal-based remedies and illnesses treated

Similarly to the results obtained by Alves and Rosa [5,6,59], Ashwell and Walston [83], Begossi and Braga [56], Branch and Silva [84], Costa Neto and Oliveira [25], El-Kamali [54] in their studies of animal-based medicines in human ethnomedicine, the interviewees in the present study cited a wide range of materials derived from animal bodies (or their metabolic products) that are used as remedies in local EVM: bone, fat/suet, feathers, heads, homemade butter, honey, horns, leather, meat, milk, rattles (from rattlesnakes), 'saburá', skulls, stingers, tails, urine, and viscera. *Nasutitermes corniger *and *Cyanocorax cyanopogon *are utilized whole.

Fats (and/or suet) is the most frequently used natural remedies, being extracted from 27 different medicinal animals. Other studies have likewise highlighted fat as one of the principal components of zootherapy in Brazil as well as in various parts of the world [5,6,21,55,59,66,68,85,86]. In Bolivia, for instance, fat is obtained from 9 of the 14 animals (including *A. paca*) used for medicinal purposes by the Tsimané Amerindians [55]. In India, fat is used in medicinal preparations for treating several ailments (e.g., burns, rheumatic and other pains) [86]. Vázquez et al. [18] identified 74 animal species used in local zootherapy in Alto dos Chiapas, Mexico, with the fatty tissue of animals being the principal resource exploited. Fat (or suet) was obtained from 12 of the 15 zootherapeutics animals reported by Barboza et al. [27] in EVM of Cubati, Brazil. Alves et al. [62] suggested that the intensive use of fat and suet as a zootherapeutic may be related to the fact that the animals used for medicinal purpose are generally vertebrates with significant amounts of fatty tissue that is easy to obtain, store, and transport.

While different methods of preparing and administering animal remedies were reported by the interviewees, patterns of applications could be perceived. Hard items like horns, leather, feathers and dermal plates were generally sun-dried, toasted, and crushed to powder, and then applied topically or administered orally. Seixas and Begossi [87] observed that toasting is a form of sterilization that helps prevent decomposition. Fat, suet, milk and honey are usually used directly as ointments or ingested, as was observed by Alves and Rosa [6], Alves et al. [14] and Silva [88]. Only one association with a medicinal plant was observed: cow's milk is mixed with 'mastruz' (*Chenopodium ambrosioides *L.) and given to the animal to drink to treat intestinal worms. Mixtures of milk and 'mastruz' are also used as ointments for treating fractures in animals.

Animals were used for treating 30 diseases or conditions in livestock and others domestic animals: burns, 'Caruara de bezerro' (omphaloarteritis), "chickens' gogo" (infectious coryza, a type of cold), colds, cracks in hooves of cattle, dermal inflammations, dermal nodules, ear problems, 'esponja de cavalo' (dermal wounds brought about by infestation of larvae of *Habronema muscae*), 'estrepes' (splinters in the skin), eye problems (especially blindness and inflammations), fever, furunculosis, inflammations in general, intestinal disorders, lesions, mastitis, "mother's body" (uterine prolapse), 'oca' (bovine gangrenous coryza), respiratory problems, retained placenta, rheumatism, snake bites, spine problems, swellings, throat problems, weakness, tick fever, wounds, and to calm angry animals.

Most zootherapeutic species are typically used to treat simple diseases, reflecting the main application forms reported previously (topical and oral). The most widely treated conditions were 'estrepes' (n = 145 citations for treatment), wounds (n = 62), eyes problems (n = 45), snake bites (n = 31), rheumatism (n = 27), and lesions (n = 26), and corroborate previous ethnoveterinary studies which indicated categories or types of similar or related diseases as the most important conditions treated by local residents [27,89-93]. Lesions, wounds, and 'estrepes' in livestock and pets are the most frequently treated conditions among livestock owners in the semi-arid region of Paraiba State for two probable reasons: (1) the symptoms are easy to diagnose and (2) these are common ailments considering that many plants in the Caatinga biome have thorns and most of the livestock are free-ranging animals susceptible to these types of injuries. Such cases have been confirmed by informants who traditionally raise animals.

'The thing most animals suffer from here are the thorns (...). When they get hurt you have to treat them quickly or they can die'. (Mr. P.F.L., 68 years old, Municipality of Monteiro)

'There are lots of (cactus species) here and that's why the animals hurt themselves a lot'. (Mr. J.J.N., 80 years old, Municipality of Sumé)

Some of the procedures employed by local experts do require considerable skill. For example, fat from the crab-eating fox (*Cerdocyon thous*), fat from Geoffroy's side-necked turtle (*Phrynops geoffroanus*), 'saburá' (fermented pollen) from Cupira bees (*Partamona seridoensis*), and butter made from cow's milk (*B. taurus*) are used in treating uterine prolapse in livestock, especially in mares and cows. In this procedure, the exteriorized uterus is washed with the zootherapeutic and quickly replaced. A similar procedure (although just involving washing the uterus with water) was reported by Antoine-Moussiaux et al. [94] among the Tuaregs in Niger for treating uterine prolapse in camels.

Local respondents have a clear understanding about the etiology of certain diseases or conditions in relation to seasonal changes. Colds (including infectious coryza of chickens), throat problems, and rheumatism (especially in cattle) occur (or are more noticeable), according to those interviewed, during the colder periods and, thus, they classify the disease as 'colds'. In fact, these diseases mentioned present in higher number or your symptoms are more noticeable in colder periods [95,96]. Perceptions of the occurrence of diseases or illnesses due to climatic seasonality are one of the characteristics of traditional cultures focused on animal husbandry. Livestock breeders believe that cattle, like people and other animals, need to maintain a balance between hot and cold in their bodies [97-99]. Large mammals are considered more resistant than people and less susceptible to environment forces of hot/cold, although animals like cattle can still suffer from health problems caused by these imbalances [100]. The essentially naturalistic hot/cold equilibrium theories are used to explain the etiology of diseases by local people in Latin America in general [101].

Regarding the number of zootherapeutics available per disease, only a few interviewees indicated that animal-based cures were prescribed to treat just a single disease (n = 14; 31.8%). For example, *Electrophorus electricus *and *Chelonoidis carbonaria *were recommended for treating wounds and 'estrepes', respectively. The species recommended for just a single veterinary purpose usually had low use-values (never above 0.09), with the exception of the Spotted Nothura with a moderate UV (0.33). However, there were many versatile species [102], a situation where different parts of a species provide material for preparing medicines used to treat several diseases. The most versatile species were: *Tupinambis merianae *(14 different veterinary uses), *Crotalus durissus *(11), *Ovis aries *(11), *Partamona seridoensis *(11), *Gallus gallus domesticus *(10), *Bos Taurus *(8), *Cerdocyon thous *(8), *Phrynops geoffroanus *(8), *Iguana iguana *(4), *Rhea americana *(4), *Rhinella schneideri *(4), *Apis mellifera *(3), *Leopardus tigrinus *(3), *Meleagris gallopavo *(3), *Melipona subnitida *(3), *Puma yagouaroundi *(3) and *Sus scrofa domesticus *(3).

Different species were found that were used to treat the same diseases or ailments. The honey from the Italian bee (*A. mellifera*), as well as honey from the 'jandaíra' bee (*M. subnitida*), the Cupira bee (*P. seridoensis*) and the 'canudo' bee (*Scaptotrigona *sp.) were all prescribed to treat eye problems, especially blindness and inflammations. The treatment of 'junta dura' (rheumatism) in cattle and equines, involves the use of fat from the South American rattlesnake, or from domestic chickens, the Gray Brocket (*Mazama gouazoupira*), suet from castrated rams or the bones of the Striped hog-nosed skunk (*Conepatus semistriatus*). The possibility of using various remedies to treat the same ailment is very popular [103], as it allows treatments to be adapted to the availability/accessibility of any of these animals in any given season [104].

### Parallels between local ethnoveterinary medicine and human ethnomedicine

One important strategy for overcoming the eventual scarcity of some traditional veterinary medicines in the semi-arid region of Paraíba State is based on the use of species that treat similar or identical ailments in humans. This link between traditional medicine and EVM exists in part because many people in NE Brazil have the habit of storing zootherapeutics medicines in their homes that are used for treating human diseases (see Alves [13], Costa-Neto [24], Ferreira et al. [28]) that can also be readily used in EVM.

We identified 19 animal species (n = 43%) that were used to treat similar ailments in both humans and animals (Table 1). For example, the suet of castrated rams (*O. aries*) was often reported to be useful in treating 'estrepes'; and according to the interviewees, this use is based on the fact that this remedy is efficient in humans. Italian bee honey was cited as a very effective remedy for curing eye problems in both humans and animals, especially inflammation and blindness. Practically all of the citations concerning the use of feathers from the Spotted Nothura to treat snake bites in livestock and pets were linked to the fact that this same remedy is useful for the same problem in humans.

Animal and human medicine have been closely linked throughout history, with each contributing to the other, and ultimately to the concept of 'one medicine' [105]. Even today, ethnoveterinary medicine and ethnomedicine overlap in many cultures, as many healers will treat humans as well as animals [106,107]. The ethnoveterinary data from the RUBIA project indicated that nearly half of the veterinary plant remedies used for mammals had similar uses in local human folk medicine [108]. About of 80% of the plants used in traditional veterinary medicine in Mediterranean Greece are used to treat similar conditions in humans [108]. Scarpa [109] reported a strong correspondence between the plants used in traditional veterinary and human medicine in the Chaco in northwestern Argentina, with 60% of the ethnoveterinary therapeutic plants having identical uses in human medicinal therapy.

The use of folk remedies to treat diseases or ailments in animals based on similar or identical illness that attack humans was denominated 'human models for animal diseases' by Barboza et al. [27]. The relationships between ethnoveterinary and human ethnomedicine can be easily explained in this perspective, as the main stock animals (e.g. cattle, sheep, goats, pigs, among others) are mammals [28], which often have health problems that are similar to humans with identical symptoms; these similarities have been noted by many different communities [110]. Huffman [111,112] observed that these similarities in the use of natural medicines are evidence that ethnoveterinary practices and human ethnomedicine have followed two main evolutionary pathways: one based on observations of self-medication in animals (zoopharmacognosy), and the other related to human folk medicine. The relationship between human and veterinary practices has been complex and mutual [108], and our results support other studies showing that there is no clear division between veterinary and human medicine in most traditional societies [27,28,113-116].

A confrontation of veterinary and human medical uses of resources presents an opportunity to analyze the ways in which both practices are interrelated in the health systems of pastoral communities [117]. Our data demonstrates that a significant part of traditional knowledge about the use of medicinal animals in Brazilian EVM is linked to traditional human medicines, and contributes to considering these practices as 'one ethnomedicine'.

### Magic-religious uses

In addition to furnishing raw materials for treating diseases, animal products are also used in the form of amulets and '*simpatias*' (popular beliefs) to prevent problems associated with natural or unnatural causes. Popular beliefs often have implications for the way species are used (either alive or dead), depending on the beliefs of a given community [7].

Ten species (seven of them wild) were linked to local beliefs in the two study sites: the domestic goat (*Capra hircus*), the crab-eating fox (*C. thous*), domestic cattle (*Bos taurus*), the White-naped Jay (*Cyanocorax cyanopogon*), the Little Tiger Cat (*Leopardus tigrinus*), the Gray brocket (*Mazama gouazoupira*), the domestic ram (*Ovis aries*), the Crab-eating raccoon (*Procyon cancrivorus*), the 'jaguarondi' (*Puma yagouaroundi*), and the Collared Anteater (*Tamandua tetradactyla*). Beliefs in local EVM can be grouped into two categories: (1) those that protect against attacks from other animals and (2) those that protect pets or livestock against the 'evil eye'.

The first category includes the use of leather of domestic goat, domestic ram and cattle, which are used to protect livestock against snake bites. In these cases, a piece of leather is 'blessed' by some local healer and then hung around the animal's neck. Also in this first category are the tail and leather of the crab-eating fox, the little tiger cat and the jaguarundi, leather from the collared anteater and the tail of the crab-eating raccoon, which are all considered amulets that can protect livestock from attacks by bats.

Zootherapeutics derived from the white-naped jay, the horns and skulls of cattle or the domestic ram, and the horns of the gray brocket compose examples of the second category of beliefs, and they are often left as amulets in places where animals are kept (corrals for livestock, sheds where pets sleep, etc.) in order to protect them from the 'evil eye'. Belief systems determine the relationships between humans and the surrounding biodiversity, and social, economic and cultural factors also play a large role in determining how individuals and communities use natural resources [118]. The interrelationships between popular beliefs and zootherapy have been studied in many different areas in Brazil [56,84,119] and have indicated that these connections must be considered in scientific studies, and when designing public health programs for communities that have traditional medical practices for both humans and animals.

Religious beliefs are important components of any culture [120] and every culture establishes positive or negative interactive connections with its environment. In addition to common uses usually deemed more important or more prominent, such as food, craft and medicinal uses, magical-religious uses of wild species of animals must be considered when developing strategies for wildlife management and conservation.

### Storage, sanitary conditions and methods of obtaining zootherapeutics species

The storage of zootherapeutic products was related to the type of resource and its consistency and durability. Medicinal products are usually stored in plastic or glass jars, while perishable products (e.g., bile, eggs, milk, meat, and viscera) were only obtained when needed. The interviewees mentioned that non-perishable ingredients, particularly hard items (e.g., bones, feathers, horns, leathers, rattles, skulls) could be stored for over a year; this same attitude was taken in regards to fat and suet. This agrees with Seixas and Begossi [87] who suggested that the use of animals as medicines might be related to the durability of the products extracted (animal fat, for example, is easily extracted and stored at room temperature and also widely used); other products that are dehydrated, toasted and ground are also easily preserved.

An important aspect noted, similar to Alves et al. [7] and Alves and Rosa [6], was that the sanitary conditions of zootherapeutic product storage were generally poor, with obvious contamination risks to these products. Practically none of the interviewees demonstrated any concern about the storage conditions (light, temperature, humidity) of animal-based medicines, and on most occasions we found little or no attention given to the way zootherapeutics were handled. For example, several livestock keepers were observed applying fat or suet over wounds without using gloves. These observations point to the need for greater sanitary precautions with medicinal animal products, and to the importance of including considerations of zootherapy into public health programs.

Organs and various tissues (including bones and bile) can be a source of *Salmonella *infections and can cause chronic diarrhea and endotoxic shock [121]. Schnurrenberger and Hubbert [122] drew attention to the possibility of transmitting serious and widespread zoonoses such as tuberculosis or rabies, and these risks should be considered whenever animal tissues from unknown sources are handled and used as remedies. *Campylobacter *and *Streptobacillus *infections or meningitis caused by bacteria of the genus *Streptococcus *can also be transmitted from animals to humans or other animals [123].

Although the need for implementing sanitary controls on the use and storage of animals and their parts for veterinary medicine purposes is clear, any proposed regulatory measures will face significant challenges - including ensuring the appropriate involvement of all users of the resources involved, active monitoring, and combating illegal, unregistered and unregulated trade or local uses. Based on Alves and Rosa [15], we suggest here some categories of regulatory measures for zootherapeutics remedies in traditional veterinary medicine: (1) More information about the risks and advantages of using medicinal animals; (2) implementation of local health programs to inform the public about the risks of zoonoses due to the inappropriate use of zootherapeutics, and (3) raise the awareness of local residents about impacts arising from the use of animals for medicinal purposes on local biodiversity. This information could be passed by public agencies (EMATER, in the case of the Paraiba State) in partnership with local farmers' associations.

The origins of the zootherapeutic products must also be inserted in the scope of Brazilian regulatory measures. Most interviewees (n = 42; 62.7%) indicated that they personally obtained the medicinal animals used in EVM in the study area. These animals were obtained in two basic manners: (1) hunting or gathering (98%) and (2) occasional trade. According to the interviewees, the animal-based remedies obtained through trade were from species not native to the Caatinga biome (allochthonous zootherapeutics); but these animals or parts were not preferred for use because of the difficulties involved in obtaining them. The prices of these purchased remedies ranged from US$0.50 (about 12 g of fat from the electric eel) to US$16.5 (for an approximately 22 cm slab of black alligator). Interviewees who bought some of their zootherapeutic medicines did not demonstrate significant concern about the veracity or origin of those products.

The hunting and gathering of wildlife species used in traditional veterinary medicine likewise highlights the need for integrating zootherapeutic practices into local animal management and conservation plans. Hunting with a shotgun is the most commonly used strategy for catching *Cariama cristata, Cavia aperea, Cerdocyon thous, Conepatus semistriatus, Coragyps atratus, Crotalus durissus, Dasypus novemcinctus, Euphractus sexcinctus, Iguana iguana, Kerodon rupestris, Leopardus tigrinus, Nothura maculosa cearensis, Puma yagouaroundi, Procyon cancrivorus *and *Tamandua tetradactyla*. Traps are also used to capture some of these species (e.g., armadillo cages and '*fôjo*' [104]), but not commonly. Shotguns have greatly facilitated the taking of specimens of the local fauna, and based on hunters stories, this is one of the most aggressive and efficient forms of harvesting. It will soon become necessary to develop sustainable strategies for using wild animals for medicinal purposes in the study area or risk the extinction of many species, as 17 of these animals are currently on the IUCN Red List (Table 1), with one near threatened and two vulnerable. Although in the studied area traditional medicine does not constitute the main form of exploitation on the local fauna, it can contribute synergistically to decrease the populations of wild species used as sources of remedies. It is often difficult to establish whether there is a threshold limit which over-exploitation of wildlife, particularly when the wildlife undergoes several types of concomitant negative impacts (e.g., habitat loss, hunting, trade/local market). Sustainability can be attained only if the exploitation of wildlife for economic, health, social and cultural purposes does not significantly affect the animal population, their habitat and the ecological function they perform [124].

## Conclusions

Our surveys in Cariri micro-region, Paraiba State, NE Brazil, revealed a rich knowledge about the uses of animals in traditional veterinary medicine. Although this knowledge is gradually being eroded, the perceived efficacy of these remedies and their economic and geographic accessibility are responsible for the continued popularity of zootherapy in the study areas.

We were able to verify that many species have wide ranges of utility, but the super-valorization and super-utilization of these animals, when associated with other practices such as ethnomedicine for humans and subsistence hunting, may raise the harvesting of these species to unsustainable levels. The most threatened medicinal species must receive special and urgent attention, and other factors such as the loss or alteration of habitats must be discussed.

Many parts of the Brazilian semi-arid region are rapidly being altered, both ecologically and culturally, and further ethnopharmacological studies will be necessary to increase our understanding of the links between traditional uses of faunal resources and conservation biology, public health policies, sustainable management of natural resources, and bio-prospecting.

## Competing interests

The authors declare that they have no competing interests.

## Authors' contributions

WMSS, JSM and RRNA - Writing of the manuscript, literature survey and interpretation; WMSS and RRDB- Ethnozoological data, literature survey and interpretation; WMSS, LCSL, RFPL and RRNA - Analysis of taxonomic aspects. LETM, MVAC, WLSV and PFGM - Literature survey and interpretation. All authors read and approved the final manuscript.

## References

[B1] Astudillo VM Zottele AC Dora F Livestock development and animal health in Latin America Bol Centr Panam Fiebre Aftosa 1991 57 15 22

[B2] Guedes PHMQ A colonização do sertão da Paraíba: agentes produtores do espaço e contatos interétnicos (1650 - 1730) Dissertação Mestrado 2006 Universidade Federal da Paraíba, Centro de Ciências Exatas e da Natureza, Programa de Pós-Graduação em Geografia

[B3] Andrade MC Szmrecsànyi T São Paulo A Pecuária e a Produção de Alimentos no Período Colonial História Econômica do Período Colonial 1996 Brasil: Editora HUCITEC - Fapesp 99 109

[B4] Ribeiro D O Povo Brasileiro: A formação e o sentido do Brasil 1995 2 São Paulo, Brasil: Companhia das Letras

[B5] Alves RRN Rosa IL Zootherapeutic practices among fishing communities in North and Northeast Brazil: A comparison J Ethnopharmacol 2007 111 82 103 10.1016/j.jep.2006.10.033 17118592

[B6] Alves RRN Rosa IL From cnidarians to mammals: The use of animals as remedies in fishing communities in NE Brazil J Ethnopharmacol 2006 107 259 276 10.1016/j.jep.2006.03.007 16621379

[B7] Alves RRN Rosa IL Santana GG The Role of Animal-derived Remedies as Complementary Medicine in Brazil BioScience 2007 57 949 955 10.1641/B571107

[B8] Almeida AV Alves AGC, Lucena RFP, Albuquerque UP Prescrições zooterápicas indígenas brasileiras nas obras de Guilherme Piso (1611-1679) Atualidades em Etnobiologia e Etnoecologia 2005 1 Recife, Brazil: Sociedade Brasileira de Etnobiologia e Etnoecologia, Nuppea 47 60

[B9] Figueiredo N Os 'bichos' que curam: os animais e a medicina 'folk' em Belém do Pará Bol Mus Para Emílio Göeldi 1994 10 75 91

[B10] Costa-Neto EM Traditional use and sale of animals as medicines in Feira de Santana City, Bahia, Brazil Ind Knowledge Dev Monitor 1999 7 6 9

[B11] Almeida CFCBR Albuquerque UP Uso e conservação de plantas e animais medicinais no Estado de Pernambuco (Nordeste do Brasil): Um estudo de caso Interciencia 2002 27 276 285

[B12] Alves RRN Santana GG Use and commercialization of Podocnemis expansa (Schweiger 1812) (Testudines: Podocnemididae) for medicinal purposes in two communities in North of Brazil J Ethnobiology Ethnomedicine 2008 4 6 10.1186/1746-4269-4-6 PMC2254592 18208597

[B13] Alves RRN Fauna used in popular medicine in Northeast Brazil J Ethnobiology Ethnomedicine 2009 5 1 30 10.1186/1746-4269-5-1 PMC2628872 19128461

[B14] Alves RRN Barbosa JAA Santos SLDX Souto WMS Barboza RRD Animal-based Remedies as Complementary Medicines in the Semi-arid Region of Northeastern Brazil Evid-Based Compl Alt 2011 2011 ID 179876 1 15 10.1093/ecam/nep134 PMC3094714 19729490

[B15] Alves RRN Rosa IL Why study the use of animal products in traditional medicines? J Ethnobiology Ethnomedicine 2005 1 1 5 10.1186/1746-4269-1-1 PMC1277085 16270931

[B16] Mahawar MM Jaroli DP Animals and their products utilized as medicines by the inhabitants surrounding the Ranthambhore National Park, India J Ethnobiology Ethnomedicine 2006 2 5 10.1186/1746-4269-2-5 PMC1636027 17081314

[B17] Mahawar MM Jaroli DP Traditional knowledge on zootherapeutic uses by the Sahari tribe of Rajasthan, India J Ethnobiology Ethnomedicine 2007 3 6 10.1186/1746-4269-3-6 PMC1892771 17547781

[B18] Vázquez PE Méndez RM Guiascón ÓGR Piñera EJN Uso medicinal de la fauna silvestre en los Altos de Chiapas, México Interciencia 2006 31 491 499

[B19] El-Hani CN Bandeira FPSF Valuing indigenous knowledge: to call it ''science'' will not help Cult Stud of Sci Educ 2008 3 751 779 10.1007/s11422-008-9129-6

[B20] Ferreira FS Brito S Ribeiro S Almeida W Alves RRN Zootherapeutics utilized by residents of the community Poco Dantas, Crato-CE, Brazil J Ethnobiology Ethnomedicine 2009 5 21 10.1186/1746-4269-5-21 PMC2731045 19656376

[B21] Alves RRN Neto NAL Brooks SE Albuquerque UP Commercialization of animal-derived remedies as complementary medicine in the semi-arid region of Northeastern Brazil J Ethnopharmacol 2009 124 600 608 10.1016/j.jep.2009.04.049 19422902

[B22] Pieroni A Giusti ME Grazzini A Fleurentin J, Pelt JM, Mazars G Animal remedies in the folk medicinal practices of the Lucca and Pistoia Provinces, Central Italy Des sources du savoir aux médicaments du futur/from the sources of knowledge to the medicines of the future 2002 1 Paris: IRD Editions 371 375

[B23] Calixto JB Twenty-five years of research on medicinal plants in Latin America: A personal view J Ethnopharmacol 2005 100 131 134 10.1016/j.jep.2005.06.004 16006081

[B24] Costa-Neto EM Healing with animals in Feira de Santana City, Bahia, Brazil J Ethnopharmacol 1999 65 225 230 10.1016/S0378-8741(98)00158-5 10404420

[B25] Costa-Neto EM Oliveira MVM Cockroach is Good for Asthma: Zootherapeutic Practices in Northeastern Brazil Human Ecol Rev 2000 7 41 51

[B26] Alves RRN Animal-Based Remedies as Complementary Medicine in Brazil Forsch Komplementmed 2008 15 226 227 18787332 10.1159/000144178

[B27] Barboza RRD Souto WMS Mourão JS The use of zootherapeutics in folk veterinary medicine in the district of Cubati, Paraíba State, Brazil J Ethnobiology Ethnomedicine 2007 3 14 10.1186/1746-4269-3-14 PMC2008192 17825094

[B28] Confessor M Mendonca L Mourao J Alves R Animals to heal animals: ethnoveterinary practices in semi-arid region, Northeastern Brazil J Ethnobiology Ethnomedicine 2009 5 37 10.1186/1746-4269-5-37 PMC2788532 19941663

[B29] McGaw LJ Van der Merwe D Eloff JN In vitro anthelmintic, antibacterial and cytotoxic effects of extracts from plants used in South African ethnoveterinary medicine Vet J 2007 173 366 372 10.1016/j.tvjl.2005.09.004 16239113

[B30] FAO Genetics and animal health-Splotlight 2002 Rome: FAO

[B31] Muhammad G Khan MZ Hussain MH Iqbal Z Iqbal M Athar M Ethnoveterinary practices of owners of pneumatic-cart pulling camels in Faisalabad City (Pakistan) J Ethnopharmacol 2005 97 241 246 10.1016/j.jep.2004.11.008 15707760

[B32] Mathias E McCorkle CM Bunders J, Haverkort B, Hiemstra W Animal health Biotechnology: Building on Farmers' Knowledge 1997 1 Basingstoke, UK: MacMillan Education Publishing 22 51

[B33] Wanzala W Zessin KH Kyule NM Baumann MPO Mathias E Hassanali A Ethnoveterinary medicine: a critical review of its evolution, perception, understanding and the way forward Livest Res Rural Dev 2005 17

[B34] Nyamanga PA Suda C Aagaard-Hansen J The socio-cultural context and practical implications of ethnoveterinary medical pluralism in western Kenya Agr Hum Values 2008 25 513 527 10.1007/s10460-008-9141-1

[B35] Lans C Turner N Khan T Brauer G Boepple W Ethnoveterinary medicines used for ruminants in British Columbia, Canada J Ethnobiology Ethnomedicine 2007 3 22 10.1186/1746-4269-3-22 PMC1831764 17324258

[B36] Alves RRN Souto WMS Ethnozoology in Brazil: current status and perspectives J Ethnobiology Ethnomedicine 2011 7 1 22 10.1186/1746-4269-7-1 PMC3166889 21767370

[B37] Rastogi S Kaphle K Sustainable Traditional Medicine: Taking the Inspirations from Ancient Veterinary Science Evid-based Compl Alt 2008 5 1 6 10.1093/ecam/nen016 PMC3094705 18980947

[B38] IBGE - Canal Cidades@ http://www.ibge.gov.br/cidadesat/default.php

[B39] Rodriguez JL Bezerra CP Magalhães CMG Telles GMVV Silva JN Carvalho MGRF Travassos MSB Maciel VdS João Pessoa Atlas Escolar da Paraíba 2002 3 Brasil: Grafset

[B40] EMBRAPA - Urbanização nos Municípios da Paraíba http://www.urbanizacao.cnpm.embrapa.br/conteudo/uf/pb.html

[B41] Brazilian Government Diagnóstico do Município de Monteiro 2005 1 Recife, Pernambuco: Ministério de Minas e Energia. Secretaria de Geologia, Mineração e Transporte Mineral

[B42] PNUD-ONU ADH - Atlas do Desenvolvimento Humano Book ADH - Atlas do Desenvolvimento Humano 2004 vol, 1.0 edn pp City

[B43] Albuquerque AW Neto FL Srinivasan VS Santos JR Manejo da cobertura do solo e de práticas conservacionistas nas perdas de solo e água em Sumé, PB Rev Bras Eng Agríc Ambient 2002 6 136 141 10.1590/S1415-43662002000100024

[B44] Huntington HP Using Traditional Ecological Knowledge in Science: Methods and Applications Ecol Appl 2000 10 1270 1274 10.1890/1051-0761(2000)010[1270:UTEKIS]2.0.CO;2

[B45] Maranhão TP Naútica e classificação ictiológica em Icaraí, Ceará: um estudo em antropologia cognitva Dissertação de Mestrado 1975 UNB

[B46] Phillips O Gentry AH Reynel C Wilkin P Galvez-Durand BC Quantitative Ethnobotany and Amazonian Conservation Conserv Bio 1994 8 225 248 10.1046/j.1523-1739.1994.08010225.x

[B47] Rossato SC Leitão-Filho HGF Begossi A Ethnobotany of caiçaras of the Atlantic Forest coast (Brazil) Econ Bot 1999 53 387 395 10.1007/BF02866716

[B48] Albuquerque UP Andrade LHC Silva ACO Use of plant resources in a seasonal dry forest (Northeastern Brazil) Acta bot bras 2005 19 27 38 10.1590/S0102-33062005000100004

[B49] Colwell RK EstimateS: Statistical estimation of species richness and shared species from samples. Version 8.2. User's Guide and application 2009 Storrs, USA http://purl.oclc.org/estimates

[B50] Colwell RK Coddington JA Estimating terrestrial biodiversity through extrapolation Phil Trans R Soc Lond B 1994 345 101 118 10.1098/rstb.1994.0091 7972351

[B51] Negi CS Palyal V Traditional Uses of Animal and Animal Products in Medicine and Rituals by the Shoka Tribes of District Pithoragarh, Uttaranchal, India Ethno-Med 2007 1 47 54

[B52] Van NDN Tap N An overview of the use of plants and animals in traditional medicine systems in Viet Nam 2008 1 Ha Noi, Viet Nam: TRAFFIC Southeast Asia, Greater Mekong Programme

[B53] Kakati LN Ao B Doulo V Indigenous Knowledge of Zootherapeutic Use of Vertebrate Origin by the Ao Tribe of Nagaland Hum Ecol 2006 19 163 167

[B54] El-Kamali HH Folk medicinal use of some animal products in Central Sudan J Ethnopharmacol 2000 72 279 282 10.1016/S0378-8741(00)00209-9 10967482

[B55] Apaza L Godoy R Wilkie D Byron EHO Leonard WL Peréz E Reyes-García V Vadez V Markets and the use of wild animals for traditional medicine: a case study among the Tsimane' Amerindians of the Bolivian rain forest J Ethnobiol 2003 23 47 64

[B56] Begossi A Braga FMS Food taboos and folk medicine among fishermen from the Tocantins River Amazoniana 1992 12 101 118

[B57] Adeola MO Importance of wild Animals and their parts in the culture, religious festivals, and traditional medicine, of Nigeria Environ Conserv 1992 19 125 134 10.1017/S0376892900030605

[B58] Costa-Neto EM Conhecimento e usos tradicionais de recursos faunísticos por uma comunidade Afro-Brasileira. Resultados preliminares Interciencia 2000 25 423 431

[B59] Alves RRN Rosa IL Zootherapy goes to town: The use of animal-based remedies in urban areas of NE and N Brazil J Ethnopharmacol 2007 113 541 555 10.1016/j.jep.2007.07.015 17719192

[B60] Moura FBP Marques JGW Zooterapia popular na Chapada Diamantina: uma Medicina incidental? Ciência & Saúde Coletiva 2008 13 2179 2188 10.1590/s1413-81232008000900023 19039402

[B61] Silva MLV Alves ÂGC Almeida AV A zooterapia no Recife (Pernambuco): uma articulação entre as práticas e a história Biotemas 2004 17 95 116

[B62] Alves RRN Lima HN Tavares MC Souto WMS Barboza RRD Vasconcellos A Animal-based remedies as complementary medicines in Santa Cruz do Capibaribe, Brazil BMC Complem Altern M 2008 8 44 10.1186/1472-6882-8-44 PMC2503950 18647413

[B63] Souto WMS Zooterápicos utilizados na Etnoveterinária nos municípios de Cubati e Pedra Lavrada, Estado da Paraíba, Brasil Graduação 2007 Universidade Estadual da Paraíba, Departamento de Biologia

[B64] Albuquerque UP Oliveira RF Is the use-impact on native caatinga species in Brazil reduced by the high species richness of medicinal plants? J Ethnopharmacol 2007 113 156 170 10.1016/j.jep.2007.05.025 17616289

[B65] Garibaldi A Turner N Cultural Keystone Species: Implications for Ecological Conservation and Restoration Ecology Soc 2004 9

[B66] Andrade JN Costa-Neto EM Primeiro registro da utilização medicinal de recursos pesqueiros na cidade de São Félix, Estado da Bahia, Brasil Acta Sci Biol Sci 2005 27 177 183

[B67] Costa-Neto EM Implications and applications of folk zootherapy in the state of Bahia, Northeastern Brazil Sust Dev 2004 12 161 174 10.1002/sd.234

[B68] Pinto AAC Maduro CB Produtos e subprodutos da medicina popular comercializados na cidade de Boa Vista, Roraima Acta Amazonica 2003 33 281 290

[B69] Alves RRN Pereira Filho GA Commercialization and use of snakes in North and Northeastern Brazil: implications for conservation and management Biodivers Conserv 2007 16 969 985 10.1007/s10531-006-9036-7

[B70] Costa-Neto EM Barata é um santo remédio: introdução à zooterapia popular no estado da Bahia 1999 1 Feira de Santana, Brazil: EdUEFS

[B71] Overview of Brazil's Legal Structure for Animal Issues http://www.animallaw.info/nonus/articles/ovbrazil.htm

[B72] Almeida AV Zooterapia indígena brasileira do século XVIII nas obras de Guilherme Piso, Georg Marcgrave e Joannes de Laet Sitientibus Série Ciências Biológicas 2007 7 261 272

[B73] Soejarto DD Biodiversity prospecting and benefit-sharing: perspectives from the field J Ethnopharmacol 1996 51 1 15 10.1016/0378-8741(95)01345-8 9213606

[B74] Lawrence EA Human and horse medicine among some Native American groups Agr Hum Values 1998 15 133 138 10.1023/A:1007435127529

[B75] Agra MF Baracho GS Nurit K Basílio IJLD Coelho VPM Medicinal and poisonous diversity of the flora of "Cariri Paraibano", Brazil J Ethnopharmacol 2007 111 383 395 10.1016/j.jep.2006.12.007 17236731

[B76] Alves RRN Silva CC Barboza RRD Souto WMS Zootherapy as an alternative therapeutic in South America J Altern M Res 2009 1 21 47

[B77] Introducing ethnoveterinary medicine http://dl.dropbox.com/u/33980395/Mathias%202001-Introducing%20ethnoveterinary%20medicine.pdf

[B78] Guèye EHF Diseases in village chickens: control through ethno-vetinary medicine ILEIA Newsletter 1997 13 20 23

[B79] Lans C Brown G Observations on ethnoveterinary medicines in Trinidad and Tobago Prev Vet Med 1998 35 125 142 10.1016/S0167-5877(97)00055-X 9646336

[B80] Iqbal Z Akhtar MS Sindhu Z-u-d Khan MN Jabbar A Herbal Dewormers in Livestock - A Traditional Therapy Int J Agr Biol 2003 5 199 206

[B81] McCorkle CM Bazalar H McCorkle CM, Mathias E, van Veen TWS Field trials in ethnoveterinary R&D: lessons from the Andes Ethnoveterinary research and development 1996 1 London: Intermediate Technology Publications 265 282

[B82] Anjaria JV McCorkle CM, Mathias E, van Veen TWS Ethnoveterinary pharmacology in India: Past, present and future Ethnoveterinary research and development 1996 1 London: Intermediate Technology Publications 265 282

[B83] Ashwell D Walston N An overview of the use and trade of plants and animals in traditional medicine systems in Cambodia 2008 1 Ha Noi, Vietnam: TRAFFIC Southeast Asia, Greater Mekong Programme

[B84] Branch L Silva MF Folk medicine in Alter do Chão, Pará, Brasil Acta Amazonica 1983 13 737 797

[B85] Rodrigues E Plants and Animals Utilized as Medicines in the Jaú National Park (JNP), Brazilian Amazon Phytother Res 2006 20 378 391 10.1002/ptr.1866 16619367

[B86] Mahawar MM Jaroli DP Traditional zootherapeutic studies in India: a review J Ethnobiology Ethnomedicine 2008 4 17 10.1186/1746-4269-4-17 PMC2516508 18634551

[B87] Seixas CS Begossi A Ethnozoology of fishing communities from Ilha Grande (Atlantic forest coast, Brazil) J Ethnobiol 2001 21 107 135

[B88] Silva AL Animais medicinais: conhecimento e uso entre as populações ribeirinhas do rio Negro, Amazonas, Brasil Bol Mus Para Emílio Göeldi 2008 3 343 357

[B89] Cox PA Balick MJ The ethnobotanical approach to drug discovery Scientific Am 1994 60 65 8023119

[B90] Kudi CA Ethno-Veterinary, Complementary and Low Cost Treatment and Management of Working Animals TAWS Workshop held; 24 April 2003 2003 Silsoe, UK. Silsoe Research Institute 10

[B91] Lans C Turner N Brauer G Lourenco G Georges K Ethnoveterinary medicines used for horses in Trinidad and in British Columbia, Canada J Ethnobiology Ethnomedicine 2006 2 20 10.1186/1746-4269-2-20 PMC1559680 16893454

[B92] McCorkle CM Mathias-Mundy E Ethnoveterinary Medicine in Africa Journal of the International African Institute 1992 62 59 93 10.2307/1160064

[B93] Viegi L Pieroni A Guarrera PM Vangelisti R A review of plants used in folk veterinary medicine in Italy as basis for a databank J Ethnopharmacol 2003 89 221 244 10.1016/j.jep.2003.08.003 14611886

[B94] Antoine-Moussiaux N Faye B Vias GF Tuareg ethnoveterinary treatments of camel diseases in Agadez area (Niger) Trop Anim Health Pro 2007 39 83 89 10.1007/s11250-007-4404-1 18318345

[B95] Paul JR The Epidemiology of Rheumatic Fever Am J Public Health 1941 31 611 618 10.2105/AJPH.31.6.611 PMC1531472 18015451

[B96] Souto WMS Animais de uso etnoveterinário no semi-árido paraibano: implicações para conservação e sustentabilidade Dissertação (Mestrado) 2009 Universidade Federal da Paraíba/Universidade Estadual da Paraíba, Programa de Pós-Graduação em Desenvolvimento e Meio Ambiente

[B97] Bernand CM Enfermedad, daño e ideología 1986 1 Quito: Abya Yala

[B98] Finerman RD Pregnancy and Childbirth in saraguro: Implications for Health Care Delivery in Southern Ecuador Med Anthropol 1982 6 269 278 10.1080/01459740.1982.9987023

[B99] Foster GM Hippocrates' Latin American Legacy: Humoral Medicine in the New World 1994 1 Langhore, PA, EUA: Gordon and Breach

[B100] Hirschkind L Sal/Manteca/Panela: Ethnoveterinary Practice in Highland Ecuador Am Anthropol 2000 102 290 302 10.1525/aa.2000.102.2.290

[B101] Green EC Etiology in human and animal ethnomedicine Agr Hum Values 1998 15 127 131 10.1023/A:1007430926620

[B102] Bonet MÀ Vallès J Ethnobotany of Montseny biosphere reserve (Catalonia, Iberian Peninsula): Plants used in veterinary medicine J Ethnopharmacol 2007 110 130 147 10.1016/j.jep.2006.09.016 17059874

[B103] Ngokwey N Home remedies and doctors' remedies in Feira (Brazil) Soc Sci Med 1995 40 1141 1153 10.1016/0277-9536(94)00241-K 7597468

[B104] Alves RRN Mendonça LET Confessor MVA Vieira WLS Lopez LCS Hunting strategies used in the semi-arid region of northeastern Brazil J Ethnobiology Ethnomedicine 2009 5 1 50 10.1186/1746-4269-5-1 PMC2678999 19386121

[B105] van Veen TWS One medicine: The dynamic relationship between animal and human medicine in history and at present Agr Hum Values 1998 15 115 120 10.1023/A:1007478809782

[B106] Mathias-Mundy E Of herbs and healers Book Of herbs and healers 1989 5 City: ILEIA 20 22 edition pp 20-22

[B107] Souto WMS Mourão JS Barboza RRD Alves RRN Parallels between zootherapeutic practices in Ethnoveterinary and Human Complementary Medicine in NE Brazil J Ethnopharmacol 2011 134 753 767 10.1016/j.jep.2011.01.041 21291986

[B108] Pieroni A Giusti ME Pasquale C Lenzarini C Censorii E Gonzáles-Tejero MR Sánchez-Rojas CP Ramiro-Gutiérrez JM Skoula M Johnson C Sarpaki A Della A Paraskeva-Hadijchambi D Hadjichambis A Hmamouchi M El-Jorhi S El-Demerdash M El-Zayat M Al-Shahaby O Houmani Z Scherazed M Circum-Mediterranean cultural heritage and medicinal plant uses in traditional animal healthcare: a field survey in eight selected areas within the RUBIA project J Ethnobiology Ethnomedicine 2006 2 12 10.1186/1746-4269-2-12 PMC1447602 16563158

[B109] Scarpa GF Medicinal plants used by the Criollos of Northwestern Argentine Chaco J Ethnopharmacol 2004 91 115 135 10.1016/j.jep.2003.12.003 15036479

[B110] Alves RRN Barboza RRD Souto WMS Katerere RD, Luseba D Plants Used in Animal Health Care in South and Latin America: An Overview Ethnoveterinary Botanical Medicine: Herbal Medicines for Animal Health 2010 1 New York, USA: CRC Press 231 256

[B111] Huffman MA Self-Medicative Behavior in the African Great Apes: An Evolutionary Perspective into the Origins of Human Traditional Medicine BioScience 2001 51 651 661 10.1641/0006-3568(2001)051[0651:SMBITA]2.0.CO;2

[B112] Huffman MA Animal self-medication and ethno-medicine: exploration and exploitation of the medicinal properties of plants Proc Nutr Soc 2003 62 371 381 10.1079/PNS2003257 14506884

[B113] Njoroge GN Bussmann RW Herbal usage and informant consensus in ethnoveterinary management of cattle diseases among the Kikuyus (Central Kenya) J Ethnopharmacol 2006 108 332 339 10.1016/j.jep.2006.05.031 16879938

[B114] Maccioni S Marchini G La Bassa Val di Magra. Collana "Liguria in parole povere" 1998 1 Genova, Itália: Sagep Ed

[B115] Maccioni S Marchini G Valli Nervia e Roja. Collana "Liguria in parole povere" 1998 1 Genova, Itália: Sagep Ed

[B116] Santillán TA Méndez JL Vázquez AM López LH La percepcion de las enfermedades de los ovinos por las mujeres Tzotiles de la region de los Altos de Chiapas, Mexico Etnoecológica 2001 5 60 74

[B117] Scarpa GF Plants employed in traditional veterinary medicine by the criollos of the Northwestern Argentine Chaco Darwiniana 2000 38 253 265

[B118] Nazarea V Rodhes R Bontoyan E Gabriela F Defining indicators which make sense to local: Intra-cultural variation in perceptions of natural resources Human Organization 1998 57 159 170

[B119] Marques JGW Pescando Pescadores: Etnoecologia abrangente no baixo São Francisco Alagoano 1995 1 São Paulo, Brazil: NUPAUB/USP

[B120] Hongmao L Zaifu X Youkai X Jinxiu W Practice of conserving plant diversity through traditional beliefs: a case study in Xishuangbanna, southwest China Biodivers Conserv 2002 11 705 713 10.1023/A:1015532230442

[B121] Still J Use of animal products in traditional Chinese medicine: environmental impact and health hazards Complement Ther Med 2003 11 118 122 10.1016/S0965-2299(03)00055-4 12801499

[B122] Schnurrenberger PR Hubbert WT An outline of the zoonoses 1981 1 Ames, IA: Iowa State University Press

[B123] Kahn LH Confronting Zoonoses, Linking Human and Veterinary Medicine Emerg Infect Dis 2006 12 556 561 16704801 10.3201/eid1204.050956 PMC3294691

[B124] Sustainable use of wildlife http://www.unep.org/iyb/pdf/Sustainableuse.pdf

